# The Results of a New Distal Protection Method in Intervention for Chronic Total Occlusion of the Superficial Femoral Artery

**DOI:** 10.4061/2009/687609

**Published:** 2009-09-01

**Authors:** Tomoko Kobayashi, Atsushi Funatsu, Emiko Ejima, Hiromi Muranishi, Makoto Utsunomiya, Kensaku Shibata, Masahiro Mizobuchi, Yoshihisa Enjoji, Shigeru Nakamura

**Affiliations:** Cardiovascular Center, Kyoto Katsura Hospital, 17 Yamada-Hirao, Nishikyo-ku, Kyoto 615-8256, Japan

## Abstract

*Aims*. To determine the efficacy of a new distal protection method in SFA CTO interventions. *Methods and Results*. From June 2003 to February 2009, ninety-two consecutive, chronic total occlusions of superficial femoral arteries were treated with catheter-based intervention using a bidirectional approach. Nine of these cases were managed with our original, distal protection method, based on symptoms, angiographic images, wire resistance, and intravascular ultrasound images. The average age was 73 years; eight patients were male. The mean occlusion length was 17.1 cm. A distal protection balloon was inserted from the retrograde sheath in the popliteal artery and placed distal to the occluded lesion after successful wire crossing. Lesion dilatation with a balloon was performed antegradely and debris was removed by 6Fr. guiding catheter. Debris was retrieved from all lesions, consisting mainly of thrombus. Where we decided not to use the distal protection method, there was no distal thromboembolism. *Conclusion*. In SFA-CTO intervention, the risk of distal embolization is 10%, which can be anticipated and eliminated by the distal protection method.

## 1. Introduction

With the advent of distal protection devices, there has been a dramatic reduction in complication rates caused by dislodged thromboembolic debris released during percutaneous interventions [1–9]. However, as there is no specific protection device for peripheral intervention, we developed an original protection method for use during intervention for high-risk, superficial femoral artery (SFA) chronic total occlusion (CTO) lesions.

The PercuSurge Guardwire (Medtronic) is an occlusive balloon wire system on a distal 0.014-inch guidewire, which is well suited for carotid stenting and high-risk coronary intervention. Embolic debris dammed proximal to the occlusion balloon is removed by catheter aspiration. One of the problems with peripheral CTO intervention is distal embolization. In certain cases, there is a huge amount of plaque and thrombus inside the SFA CTO lesion. Debris from the CTO lesion is sometimes too large to be removed by the commercially available aspiration catheter, which is a 0.014-inch system. A second problem is that delivering the protection devices through the CTO lesion is difficult if the lesion is long. To resolve these issues, we have developed an original method of distal protection. In high-risk SFA-CTO cases, we deployed a distal occlusion balloon via the popliteal artery and used a 6Fr. large-lumen guiding catheter as an antegrade aspiration device.

## 2. Methods

From June 2003 to February 2009, 126 consecutive SFA CTO lesions were treated by catheter-based intervention. A bidirectional approach was employed in 92 patients (73%). In all patients, dual antiplatelet therapy was administered, aspirin 100 mg/day and ticlopidine 200 mg/day, given for more than three days before the intervention.

After successful wire crossing of the CTO lesion, an intravascular ultrasound (IVUS) study was performed before full dilatation of the lesion, to determine plaque characteristics. We decided to apply distal protection methods based on the criteria in Table 1.

When the lesion is judged as carrying a high risk of distal embolization, the popliteal artery is blocked by an angioplasty balloon or an occlusion balloon from a retrograde sheath. After the popliteal artery occlusion has been established, the lesion is fully dilated with an antegrade balloon. Debris is dammed up at the retrogradely placed occlusion balloon. The antegrade balloon is removed and a 6Fr. guiding catheter with a Y connector is advanced into the CTO lesion, just proximal to the retrograde protection balloon. Aspirating with a 30 mL syringe through the side arm of the Y connector, paying attention not to suck air through the O ring, we remove the catheter from the sheath. Debris is checked for by flushing the catheter once it has been removed. This aspiration technique is repeated until no further debris is removed.

The IVUS study is repeated and a decision is made whether stent deployment is required or not. We select the stent size and length based on IVUS findings. The landing position of the stent is selected as the portion where there is little plaque and therefore a low risk of edge dissection; we mark the position of the IVUS probe using fluoroscopy to localize the site precisely. The stent is delivered in an antegrade fashion. To check the distal stent position, contrast agent is injected through the wire lumen of the retrograde occlusion balloon. After stent deployment and optimization by balloon dilatation, debris aspiration is performed through the antegrade sheath with the guiding catheter. When no distal debris remains, the occlusion balloon is deflated and final injection via the proximal sheath is performed.

## 3. A Case Example

**Figure 1:**
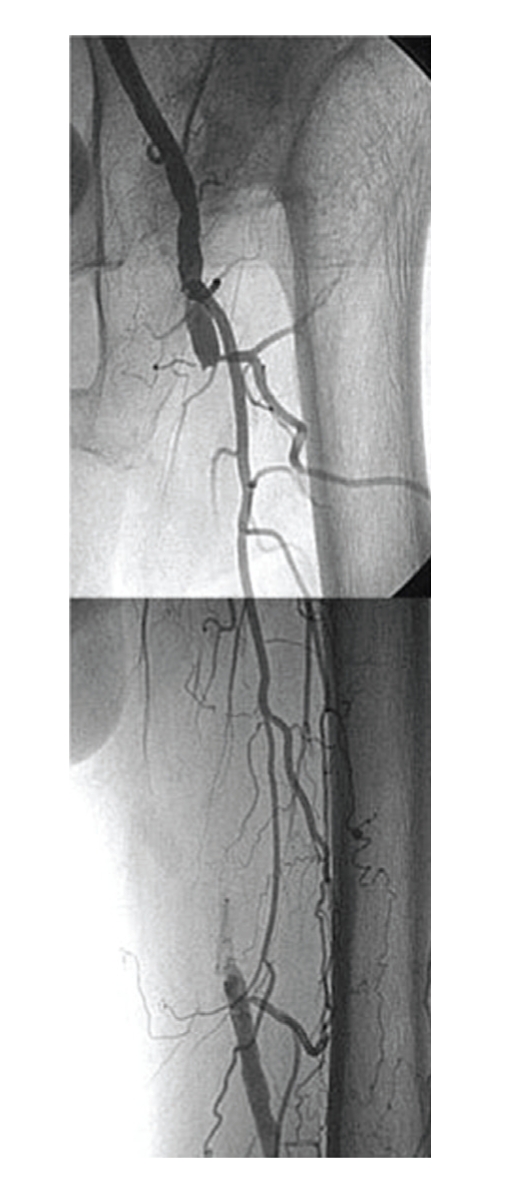
Preprocedural angiogram showed occlusion of the superficial femoral artery. The contrast agent was hazy at the distal end.

An eighty-two-year-old male with severe intermittent claudication of the left foot, Fontaine classification stage IIb, had diabetes mellitus that was controlled by oral agents. He was a current smoker. Arterial Doppler examination revealed an ankle-brachial index (ABI) of 1.05 on the right and 0.71 on the left. Diagnostic angiography showed complete occlusion (occlusion length 12 cm) of the left SFA and reconstitution above the knee at the popliteal artery, which was filled by collaterals (Figure 1).

## 4. Procedure

**Figure 2:**
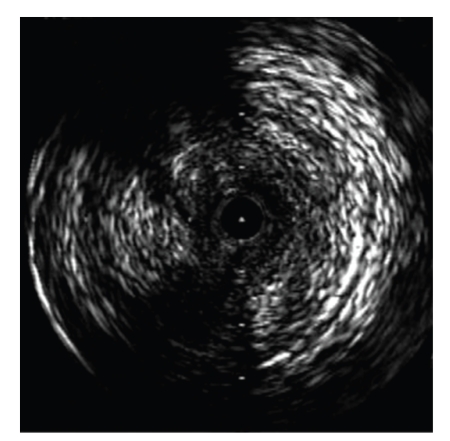
An IVUS image showed that the lesion was occupied with mixed plaque.

**Figure 3:**
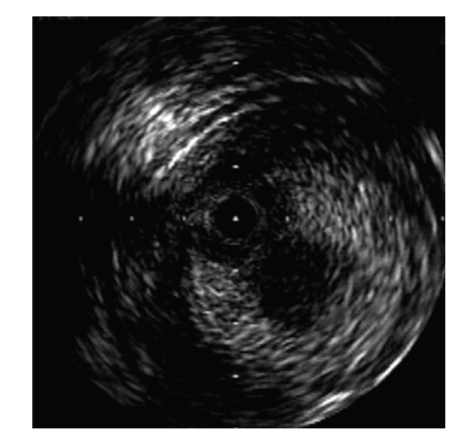
On IVUS the plaque consisted of two layers. The center of the plaque, a layer of low echogenicity, was mobile.

**Figure 4:**
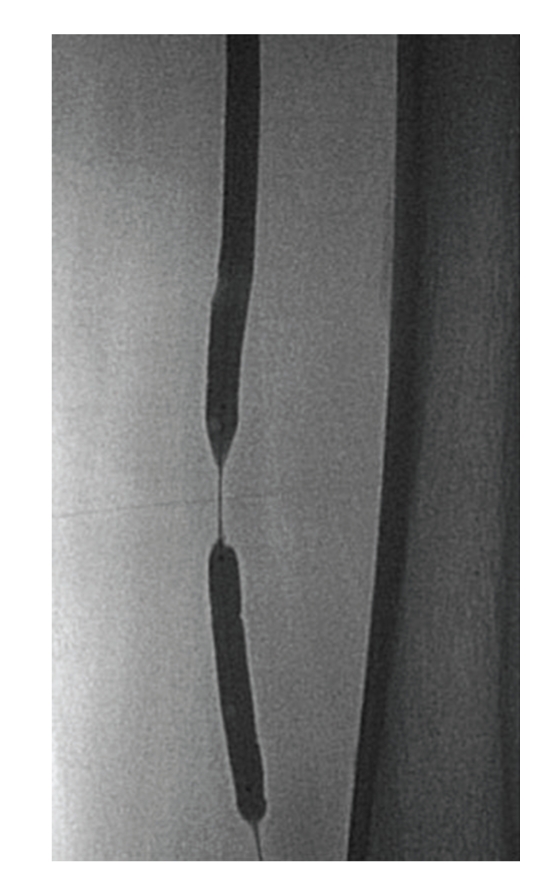
The distal PTA balloon was inserted using a retrograde approach and inflated to protect against thromboemboli. The proximal PTA balloon was inserted antegrade and used to dilate the occlusion site.

**Figure 5:**
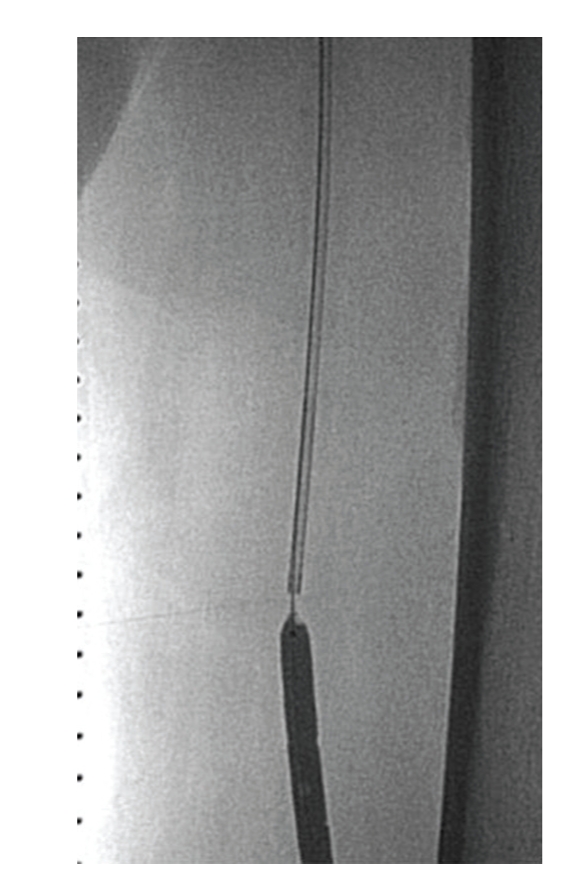
The catheter was inserted and used to suck up debris.

**Figure 6:**
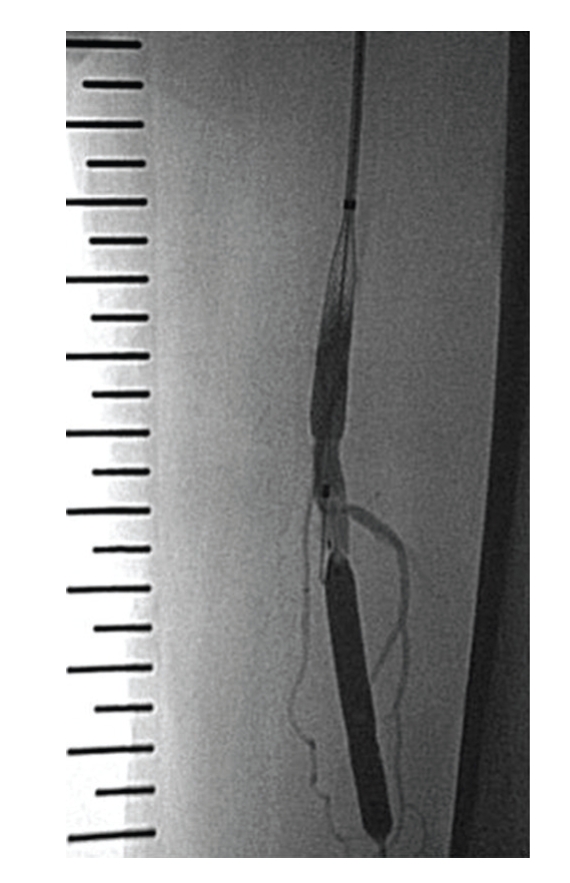
The self-expanding stents were implanted.

**Figure 7:**
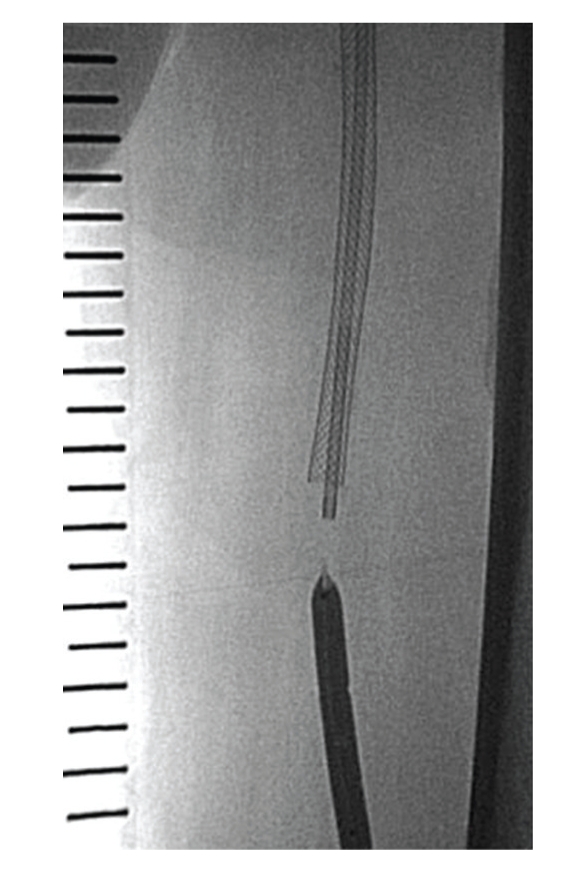
After postdilation, debris was sucked out again.

**Figure 8:**
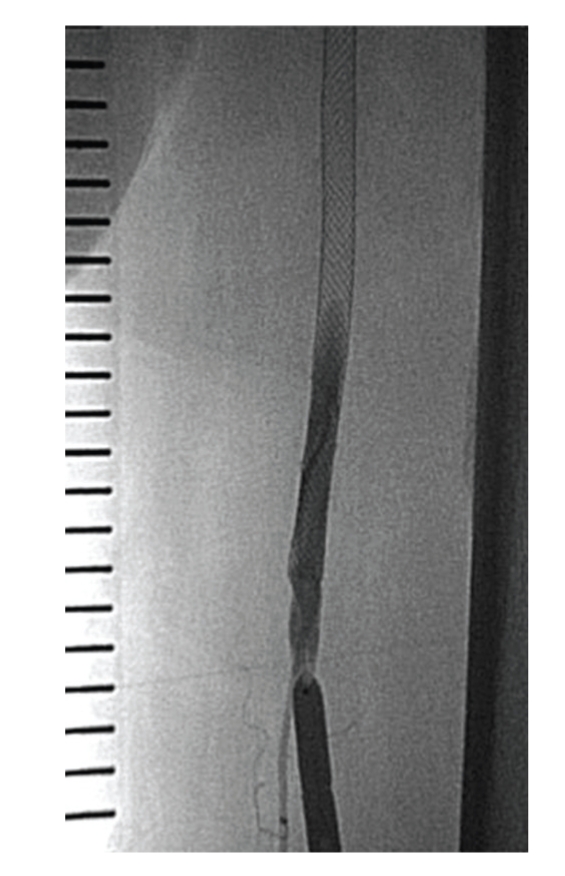
We checked for the absence of debris at the distal end of the CTO lesion by injecting dye through the tip of the lumen of the occlusion.

**Figure 9:**
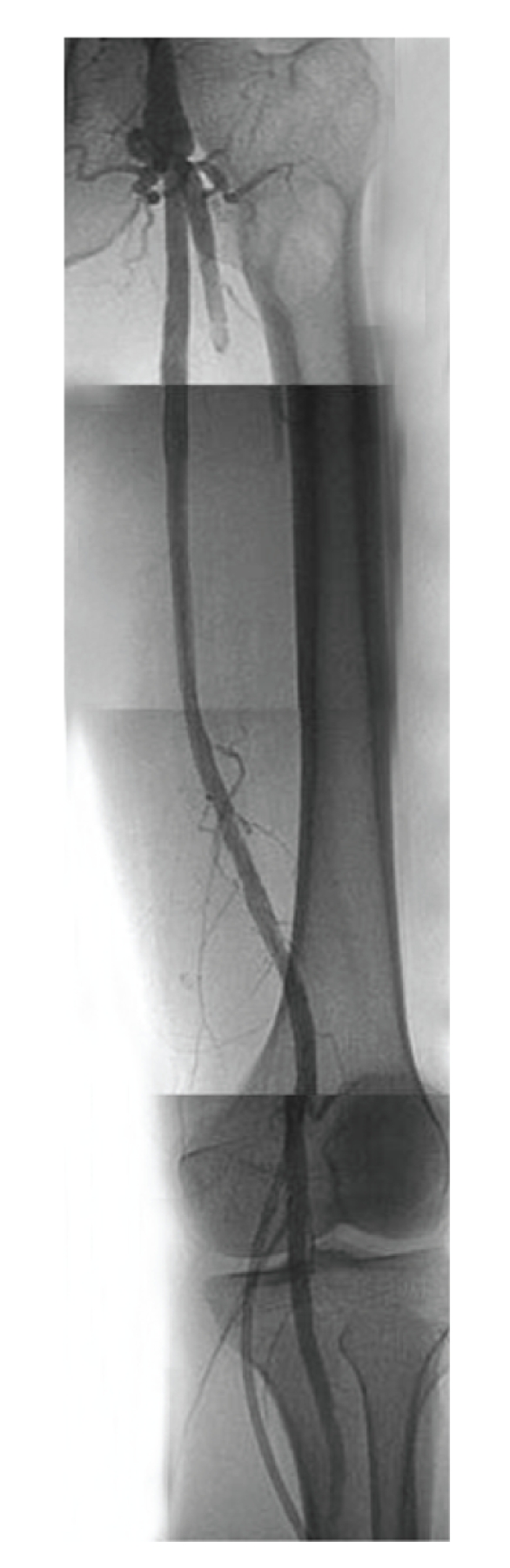
The final angiogram showed good runoff and no distal embolism.

**Figure 10:**
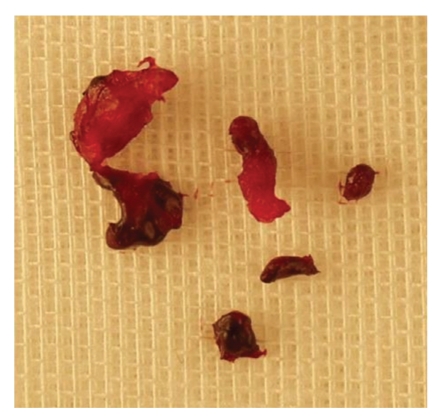
Specimen retrieved from the catheter.

**Figure 11:**
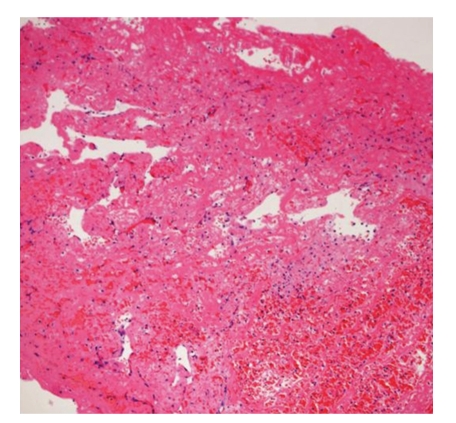
Hematoxylin and eosin stain of debris showed fibrin thrombus with inflammatory cells and immature endothelial cells.

For the retrograde approach, a 6Fr. sheath was inserted into the left popliteal artery with ultrasound guidance to prevent venous puncture. The patient was rolled over from the prone position to the supine position. A 6Fr. sheath was inserted into the left femoral artery in the antegrade direction. Through the sheath in the popliteal artery, a 0.018-inch guidewire (Treasure 12 g, Asahi Intecc) was advanced into the left SFA. The wire crossed the CTO lesion relatively quickly and emerged from the femoral sheath. The wire was exchanged for a 0.014-inch guidewire using a microcatheter (Transit, Cordis), and we examined the lesion by IVUS (Intrafocus, Terumo). IVUS imaging showed that the CTO lesion was filled with plaque of mixed echogenicity (Figures 2 and 3), implying the presence of thrombus. Based on these results, we decided to use distal protection. A 6 × 40 mm PTA balloon catheter (Opera, Abbott vascular) was inserted via the popliteal sheath and dilated at 3 atmospheres to establish distal arterial occlusion. After predilating with a 6 × 80 mm PTA balloon catheter (Submarine, INVAtec) from the left femoral sheath (Figure 4), a 6Fr. 55 cm straight guiding catheter (Autobahn, inner diameter 0.073-inch, Nipro) was advanced and plaque and thrombus were sucked from the vessel (Figure 5). We deployed an 8 × 66 mm self-expanding stent (Wall, Boston Scientific) and after dilatation was performed with a 6 × 80 mm balloon at 5 atmospheres (Figure 6). After we sucked up debris three times (Figure 7), we checked for the absence of debris at the distal end of the CTO lesion by injecting dye through the tip of the lumen of the occlusion balloon (Figure 8). Final angiography, once the protective balloon was deflated, showed good distal runoff without embolism (Figure 9). Histopathological examination of the debris showed fibrin thrombus with inflammatory cells (Figures 10 and 11). After intervention, the ABI on the left rose to 0.91 and symptoms were relieved.

## 5. Results

In nine of 92 cases (9.8%), the distal protection method was applied in management of SFA CTO procedures.

Table 2 shows the characteristics of these nine patients, the sites used for intervention and the lengths of the occluded lesions. The average age was 73 years (62–83) and eight (89%) were male.

Two patients (nos. 2 and 4) showed critical limb ischemia. The retrograde approach site was the popliteal artery. The average CTO length was 17.1 cm (6–24 cm).

Table 3 shows the interventional procedures and acute results. In six procedures, distal protection was performed using percutaneous transluminal angioplasty balloons, and in the other case, an occlusion balloon was used. All interventions (balloon angioplasty: 1, self-expanding stent: 8) were technically successful. Debris was retrieved from all lesions, consisting mainly of thrombus. In three cases (33%), distal embolism occurred to the arteries below the knee. Distally embolized thrombi were removed using a 5Fr. catheter and a monorail aspiration catheter designed for coronary artery interventions (TVAC, Nipro). After further repeated aspiration, flow to the toe recovered completely and features of limb ischemia did not appear. However, there was no case in which distal emboli occurred (0/83) after we decided that distal protection was not required. An inhospital, major adverse event occurred in one case (patient no. 2). As he had gangrene below his right ankle prior to admission, we could not avoid amputation of his right leg below the knee, one month after intervention. In all other patients, both symptoms and ABI improved.

## 6. Discussion

In cases with SFA CTO and nonacute limb ischemia, it is difficult to cross the lesion with a guidewire. However when PTA is successful, distal embolism is rare. There have been several published reports of distal embolization during lower extremity intervention. The frequency of distal embolization during thrombolytic therapy for limb-threatening ischemia is high. One published study reported an immediate distal thromboembolic rate of 5.3%, with similar results for acute and subacute versus chronically occluded lesions [10]. A report on peri-operative distal embolization following intervention for chronic iliac artery occlusion gives a rate of 2.9% [11]. Some reports on distal embolization during intra-arterial thrombolysis have reported rates of 8.3–24% [12, 13]. A report on the use of the Rotarex catheter for acute and subacute femoro-popliteal artery occlusions indicates that distal embolization during recanalization occurred in 24% of cases [14].

Angioplasty for acute limb ischemia carries a risk of progressive ischemia and tissue necrosis if distal thromboembolism occurs. A recently occluded lesion, which we call “unstable limb ischemia”, may be complicated by distal embolism during intervention, which may result in worsening ischemic symptoms. It is important to identify the high-risk group. If there is a risk of distal embolization, we believe it is best to use a method of embolic protection. It has been reported that distal protection devices, such as a guidewire with a balloon/filter and a balloon protection sheath, were beneficial in treating high-risk lesions of peripheral vascular disease [15, 16]. Another report on distal protection during femoro-popliteal atherectomy states that the filter wires retrieved debris after atherectomy in all cases and prevented distal embolic occlusion [17]. We identify vulnerability of the lesion by means of symptoms, an angiogram, resistance on wire manipulation, and IVUS imaging. Symptoms in cases with unstable limb ischemia are worsening claudication, cyanosis, ulceration, and gangrene that have occurred within a few months. The angiographic image of a case with unstable limb ischemia shows abrupt occlusion, contrast agent oozing through, or mobile clot at the origin of the occlusion. When we advance the wire into the vulnerable, occluded lesion, we feel resistance at entry but the wire easily passes through the CTO lesion. IVUS images showing mobile plaque or plaque of low echogenicity imply that the lesion is occupied by thrombus or soft plaque. A homogeneous, highly echogenic mass with small blood flow channels within the plaque also implies an unstable lesion. When we detect these findings, we apply distal protection. Because the intensity of the speckled ultrasonic signal is positively correlated with the amount of red blood cells in the thrombus [18] and the echogenicity of thrombus changes with age, the images of vulnerable plaques vary. Four features were helpful in identifying a lesion at high-risk of distal embolization. This occlusion method has the merit that it is not necessary to advance the protection device through the totally occluded lesion. We performed interventions using a bidirectional approach in complex CTO lesions. This method makes it easy to deliver the protection devices and prevent distal migration of debris. Recently we chose to use occlusion balloons via a retrograde approach, as these balloons are cheaper than PTA balloons. In our limited experience of just nine cases (10%) of SFA CTO with a distal protection method, we could remove thrombotic plaque but encountered distal emboli in 3 cases. In the remaining 83 cases in which we did not use protection, as we thought there was a low risk of distal embolization, there was no instance of distal embolism. This study demonstrates that more than 10% of SFA CTO catheter intervention is associated with a risk of distal embolization. Balloon protection method has some dead space between the occlusion balloon edge and arterial wall. If the small plaque drops down into this space, aspiration with the guiding catheter cannot remove plaque completely. In our 3 cases we dropped small debris into the distal artery below the knee and needed additional procedures to rescue limb ischemia.

This study had several limitations. First, the sample size is small. Second, it is difficult to validate our methods used to identify an unstable plaque. Third, our methods cannot completely prevent distal embolism because all of the debris cannot be aspirated through the guiding catheter.

## 7. Conclusion

The method of distal protection that we describe is beneficial in treating high-risk lesions that potentially contain residual thrombus, in cases with ischemic limbs. In SFA-CTO intervention, the risk of distal embolization is 10% which can be determined based on clinical, procedural, and IVUS information.

## Figures and Tables

**Table 1:** Criteria for distal protection methods. IVUS: intravascular ultrasound.

(i) Worsening symptoms and signs
* *(1) Newly onset (<6 months) of claudication
* *(2) Shortend walking distance
* *(3) Pain at rest
* *(4) Ulceration or gangrene of extremities

(ii) Angiographic features
* *(1) Contrast stain at the occlusion site
* *(2) Contrast defect due to thrombus

(iii) Mild resistance on the wire manipulation
* * * *Easy pass through the CTO lesion by a guidewire

(iv) IVUS findings
* *(1) Poorly echogenic plaque
* *(2) Mobile plaque
* *(3) Homogeneous plaque of mildly raised echogenicity
* *(4) Plaque with small blood flow channels

**Table 2:** Patient characteristics, approach site and occlusion length. Pt: patient, F: female, M: male, HT: hypertention, DM: diabetes, IC: intermittent claudication, CLI: critical limb ischemia, Rt: right, Lt: left, SFA: superficial femoral artery, FA: femoral artery, PA: popliteal artery.

Pt. no	Age	sex	Risk factor	Diagnosis (Fontaine)	Target SFA	Antegrade-approach	Petrograde-approach	Occlusion length
1	83	F	HT	IC (IIb)	Rt	Lt FA 6F	Rt PA 6F	16.5 cm
2	74	M	HT, DM, smoking	CLI	Rt	Rt FA 7F	Rt PA 4F	15 cm
3	82	M	DM, smoking	IC (IIb)	Lt	Lt FA 6F	Lt PA 6F	12 cm
4	72	M	HT, smoking	CLI	Rt	Lt FA 6F	Rt PA 4F	17 cm
5	62	M	HT, smoking	IC (IIa)	Lt	Lt FA 6F	Lt PA 6F	23 cm
6	83	M	HT, smoking	IC (IIb)	Lt	Lt FA 6F	Lt PA 4F	18 cm
7	70	M	HT, smoking	IC (IIb)	Rt	Rt FA 6F	Rt PA 6F	6 cm
8	65	M	HT, smoking	IC (IIb)	Rt	Lt FA 6F	Rt PA 6F	22 cm
9	62	M	HT, DM, Dyslipidemia	IC (IIb)	Rt	Rt FA 6F	Rt PA 6F	24 cm

**Table 3:** Procedure and results. ABI: ankle and brachial index.

Pt. no	Protection device	Intervention device	Success	ABI (Pre–post)	Debris	Distal embolism	Pathology of debris
1	PTA balloon	Balloon	yes	0.6–0.83	+	−	Thrombus with hyalinization and inflammatory cells

2	PTA balloon	Stent	yes	0–none	+	−	Fibrin thrombus

3	PTA balloon	Stent	yes	0.71–0.91	+	−	Fibrin thrombus with inflammatory cells

4	PTA balloon	Stent	yes	0–1.01	+	−	Fresh thrombus

5	Occlusion catheter	Stent	yes	0.57–0.9	+	−	Fibrin thrombus with neutrophils

6	PTA balloon	Stent	yes	0–1.21	+	+	Fibrin thrombus

7	Occlusion catheter	Stent	yes	0.54–1.34	+	+	Fibrin thrombus

8	Occlusion catheter	Stent	yes	0.76–1.04	+	+	Fibrin thrombus

9	PTA balloon	Stent	yes	0.63–1.04	+	−	Fibrin thrombus
